# Conserved and non-conserved enhancers direct tissue specific transcription in ancient germ layer specific developmental control genes

**DOI:** 10.1186/1471-213X-11-63

**Published:** 2011-10-20

**Authors:** Sumantra Chatterjee, Guillaume Bourque, Thomas Lufkin

**Affiliations:** 1 Stem Cell and Developmental Biology, Genome Institute of Singapore, 60 Biopolis Street, 138672, Singapore; 2 Department of Biological Sciences, National University of Singapore, 117543, Singapore; 3 Computational and Mathematical Biology, Genome Institute of Singapore, 60 Biopolis Street, 138672, Singapore

## Abstract

**Background:**

Identifying DNA sequences (enhancers) that direct the precise spatial and temporal expression of developmental control genes remains a significant challenge in the annotation of vertebrate genomes. Locating these sequences, which in many cases lie at a great distance from the transcription start site, has been a major obstacle in deciphering gene regulation. Coupling of comparative genomics with functional validation to locate such regulatory elements has been a successful method in locating many such regulatory elements. But most of these studies looked either at a single gene only or the whole genome without focusing on any particular process. The pressing need is to integrate the tools of comparative genomics with knowledge of developmental biology to validate enhancers for developmental transcription factors in greater detail

**Results:**

Our results show that near four different genes (*nkx3.2, pax9, otx1b and foxa2*) in zebrafish, only 20-30% of highly conserved DNA sequences can act as developmental enhancers irrespective of the tissue the gene expresses in. We find that some genes also have multiple conserved enhancers expressing in the same tissue at the same or different time points in development. We also located non-conserved enhancers for two of the genes (*pax9 and otx1b*). Our modified Bacterial artificial chromosome (BACs) studies for these 4 genes revealed that many of these enhancers work in a synergistic fashion, which cannot be captured by individual DNA constructs and are not conserved at the sequence level. Our detailed biochemical and transgenic analysis revealed Foxa1 binds to the *otx1b *non-conserved enhancer to direct its activity in forebrain and otic vesicle of zebrafish at 24 hpf.

**Conclusion:**

Our results clearly indicate that high level of functional conservation of genes is not necessarily associated with sequence conservation of its regulatory elements. Moreover certain non conserved DNA elements might have role in gene regulation. The need is to bring together multiple approaches to bear upon individual genes to decipher all its regulatory elements.

## Background

One of the paradigms of development is the regulation of the genome in a precise and synchronized manner to form a highly complex embryo with diverse and specialized cell types. Though the major cell types in the embryo contain the same genetic material, they are very different from each other in both morphology as well as function. The generation of this cellular diversity by the genome is controlled by *cis-*regulatory elements. *Cis-*regulatory elements are DNA elements that are a key component of the genome's non-coding functional sequences and consist of promoters, enhancers, silencers, insulators and locus control regions (LCR). The idea that animal development is regulated by *cis*-regulatory DNA elements is well established and has been elegantly described in invertebrates [1-3]. Of the many *cis-*regulatory elements, enhancers are critical in modulating tissue specific and time dependent gene expression during embryonic development [4-7]. Enhancers are thought to consist of clustered target sites for a number of transcription factors and collectively form the genomic instructions for developmental gene regulatory networks. Hence any approach to elucidate such networks necessitates the discovery of all constituent enhancer elements and their genomic locations.

The identification and characterization of such *cis-*regulatory regions within the non-coding region of vertebrate genomes remains a challenge for the post genomics era. Traditionally enhancers have been identified through deletion assays [8,9] and *in vitro *footprinting [10,11]. But recent advances in sequencing coupled with improved alignment tools have made comparative genomics one of the favored methods for enhancer detection [12-15].

It is suggested that 1% of the human non-coding genome is at least 70% conserved in the mouse genome over a region of 100 bp or longer [16,17]. Many such conserved non-coding elements (CNEs) lie next to critical developmental control genes and have been shown to be developmental enhancers when selected and tested in mice and zebrafish [14,18,19]. It has also been demonstrated that genomic regions that have no sequence level conservation among different species can act as robust developmental enhancers [13,20]. Recently chromatin immunoprecipitation followed by massive sequencing (ChIP-Seq) for P300 and ChIP followed by microarray (ChIP-Chip) on histone modifying mark H3K4me3 (Histone 3 Lysine 4 trimethylation) has been shown to be useful in identifying tissue specific enhancers on a global scale, many of which have very weak sequence conservation [21-24]. Though such studies are a valuable resource they are limited by both the amount of material generated from a particular tissue as well as the availability of a specific antibody. It is also interesting to note that all enhancers are not marked by specific histone marks or co-activator proteins.

Our study takes a "gene centric" approach to locate and validate CNEs as transcriptional enhancers, thus helping to link an enhancer to gene expression and helping to understand transcriptional control of specific genes in greater depth. We looked for CNEs to validate as enhancers around four critical developmental control genes that are expressed in tissues that are ancient in origin and conserved in evolution. We sought to investigate if genes, irrespective of which germ layer they expressed in, followed the general trend of having both sequence conserved and non-conserved enhancers or if there was a bias depending on the germ layer. We picked one gene expressing in ectoderm (*otx1b*), one expressing in endoderm (*foxa2*) and two from mesoderm *(nkx3.2 *and *pax9*). As mesoderm is considered to be of more recent origin [25,26], we chose 2 genes expressing in this particular germ layer to assess whether there was any bias in enhancer conservation due to evolutionary time. We hypothesized that using stringent conservation criteria over large phylogenetic distances will lead to a better filtering out of the functional enhancers from non-functional CNEs.

Using modified Bacterial artificial chromosomes (BACs) to detect regulatory elements is highly beneficial when testing for multiple regulatory domains from noncontiguous DNA, which act in concert to regulate expression of a gene [27,28]. It has also helped in cases where when the non-coding regulatory DNA is not conserved across species and thus not recognizable prior to testing. The success of this approach lies in fact that for genes with multiple expression domains, most of these domains can be observed in a single transgenic embryo as opposed to screening for multiple embryos for individual DNA constructs. Hence, we also modified the BACs containing the 4 genes of interest to locate and validate *cis*-regulatory elements, which are not conserved at the sequence level.

## Results

### *nkx3.2*

**Figure 1:**
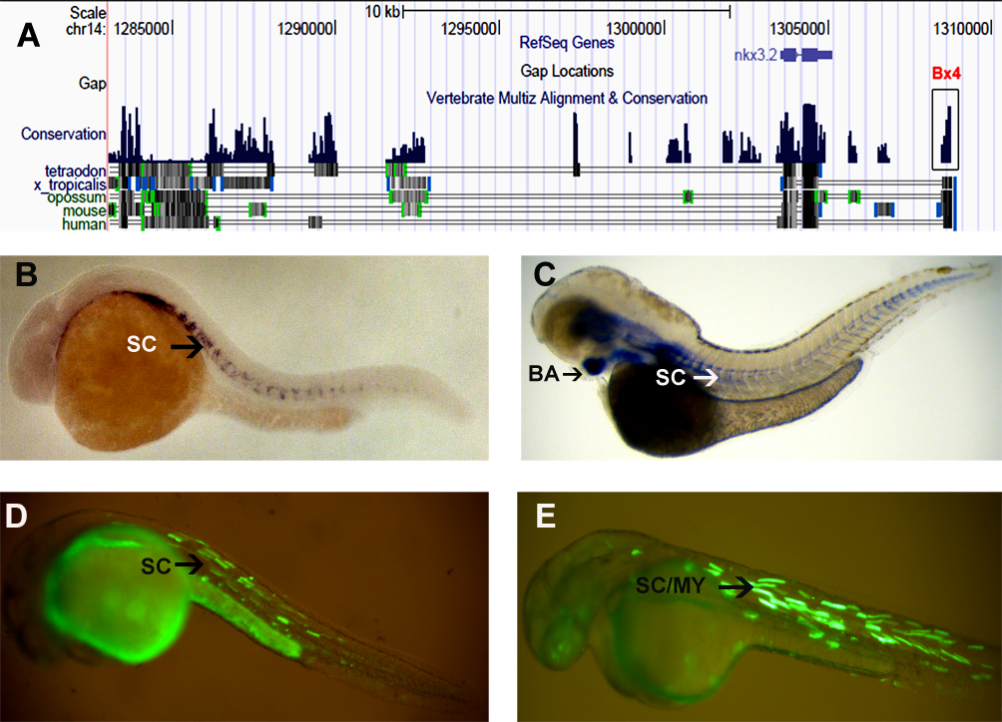
***nkx3.2 *CNEs and modified BAC**. (A) UCSC browser snapshot showing the synteny around *nkx3.2 *and the functional CNE Bx4. (B) Whole mount RNA in situ hybridization showing expression of *nkx3.2 *in the sclerotome (SC) at 24 hpf. (C) Whole mount RNA in situ hybridization showing expression of *nkx3.2 *showing expression in branchial arch (BA) and sclerotome (SC) at 48 hpf. (D) Functional CNE Bx4 drives expression of EGFP in sclerotome (SC) at 24 hpf. (E) Modified BAC shows expression in both sclerotome and myotome (MY) at 24 hpf.

*Bagpipe *related homeobox containing genes are members of the ancient *NK *gene family and are highly conserved in sequence and function from *Drosophila *to humans. In mammals it restricts the multipotential mesodermal progenitor to a chondroblast lineage and helps in the development and evolution of the axial skeleton in mouse and has potential role in human skeletal disorders [29-32]. It also has a known role in jaw joint formation and patterning [31,32]. *nkx3.2 *has also been detected in the dorsal and anal fin radials of zebrafish as late as 10 dpf and in the distal chondrocytes [33]. We detected *nkx3.2 *expression by RNA *in situ *in the sclerotome and parts of anterior branchial arches at 24 and 48 hpf (Figure 1B, C).

### CNEs in and around *nkx3.2*

UCSC genome browser's alignment of sequences near *Nkx3.2 *in zebrafish revealed four conserved elements around the gene matching our criterion (≥ 100 bp length, ≥ 60% conservation) named Bx1 to Bx4 and varied in length from 318 to 990 bp (Figure 1A and Additional File 1). These pieces were co-injected with the minimal promoter-EGFP construct into 1-cell zebrafish embryos and observed for EGFP expression in the domains of expression of *nkx3.2 *in zebrafish at different time points. Only one DNA element, Bx4 drove EGFP expression that was detected along the antero-posterior axis in the region of the emerging sclerotome and myotome at 48 hpf (Figure 1D and Additional File 2). Because transient transgenics in zebrafish tend to produce mosaic expression we did immunohistochemistry with an EGFP antibody to detect the exact expression domain of this enhancer as previously described [34]. Comparison with the RNA *in situ *data at the time point revealed the expression of the enhancer matched that of the endogenous gene (Additional File 3). There was no EGFP expression from the remaining CNEs at the developmental time points under observation. For this gene we also cloned all the four enhancer elements upstream of the promoter in the same order as they are found in the genome and this "mega" construct was also tested. We did not see any significant increase in expression level or domain from all the four enhancers versus only the single functional CNE (data not shown). Thus only 1 out of the 4 CNEs functioned as a developmental enhancer at all time points under observation.

### *nkx3.2 *BAC modification

A 186 kb BAC (CH73-353E16) containing the zebrafish *nkx3.2*gene was identified from the UCSC genome browser and modified by inserting the EGFP ORF and a selection cassette was inserted just upstream of the translational start site of the gene (Additional File 4). This ensured that the all the *cis-*regulatory elements in the 186 kb genomic region around *Nkx3.2 *that was cloned into the BAC were captured in the assay. This modified BAC was injected into 1-cell zebrafish embryos and the embryos were followed over its development to detect EGFP in domains of expression of *nkx3.2*. 70% of the embryos gave distinct expression along the antero-posterior axis as marked by sclerotome/myotome, at 24 and 48 hpf (Figure 1E), which correlated with the *nkx3.2 *expression as detected by the RNA *in situ *(Figure 1C, D). We were however unable to detect any EGFP expression in the branchial arch, which is also a domain of expression for the gene. This led us to conclude that the regulatory elements for this domain lay outside the 186 kb genomic DNA present in the BAC.

### *pax9*

**Figure 2:**
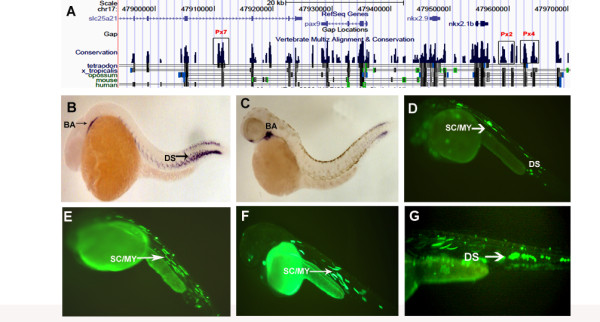
***pax9 *CNEs and modified BAC**. (A) UCSC browser snapshot showing synteny around *pax9 *and the functional CNEs Px2, Px4 and Px7. (B) Whole mount RNA in situ hybridization showing expression of *pax9 *in branchial arch and dorsal sclerotome at 36 hpf and (C) 48 hpf. (D) Functional CNE Px2 drives expression of EGFP in sclerotome/myotome (SC/MY) as well as dorsal sclerotome at 36 hpf. Enhancers Px4 (E) and Px7 (F) drive expression in the sclerotome and myotome at 36 hpf and 48 hpf respectively. (G) Modified BAC showing expression in the dorsal sclerotome at 36 hpf. BA - branchial arch. DS - dorsal sclerotome

*pax9 *is a member of the paired box (*PAX*) family of transcription factors. Members of this gene family typically contain a paired box domain, an octapeptide, and a paired-type homeodomain. The paired domain consisting of 125-128 amino acids, encoded by the paired box, was named after the *Drosophila *pair-rule segmentation gene *paired *(*prd*) where it was first identified. There are nine known P*ax *genes in mouse (*Pax1 to Pax9*) and also humans (*PAX1 *to *PAX9*). Except *Pax4 *all other *Pax *genes also are present in zebrafish. In teleost fish there is evidence to suggest that *Pax9 *is indispensable for the development of the sclerotome and the neural arch [35]. Zebrafish *pax9 *expression is initiated at the end of the segmentation period in mesenchymal sclerotome cells on both sides of the notochord. Our RNA *in situ *hybridization detected expression of *pax9 *in the dorsal sclerotome and parts of branchial arches at 36 hpf (Figure 2B) and at 48 hpf (Figure 2C)

### CNEs in and around *pax9*

Alignment of the sequences around *pax9 *revealed 11 conserved elements matching our criteria ranging in size from 311 bp to 1.2 kb (Figure 2A and Additional File 1) until there was a synteny break in one or both species that were used in the alignment. Six of these pieces were at the 5' end of the gene while five were from the 3' end, with the furthest being 178 kb away. Three out of these CNEs faithfully recaptured EGFP expression along the A-P axis as marked by the sclerotome/myotome when co-injected with the minimal promoter-reporter construct. All the three CNEs drove expression of EGFP along the presumptive sclerotome and myotome at different time points in development. Elements Px2 and Px4 which were 23 and 27 kb away from the gene on the 5' end drove expression in the sclerotome and myotome at 36 hpf (Figure 2D, E and Additional File 2), whereas Px7 which was 17 kb away from the gene at the 3' end drove EGFP expression in the same domain at 48 hpf (Figure 2F and Additional File 2). Thus 27% of the CNEs around *pax9 *were functional in our assay for enhancers.

Since all the three functional CNEs were expressing in the cells along the antero-posterior axis which contain both the sclerotome and myotome, we did immunohistochemistry for EGFP as described previously on the transgenic fish to ascertain the exact tissue which these enhancers were active in. Ours results show that all the three enhancers are expressing in the subset of sclerotomal cells which also expresses pax9 as seen by RNA in situ hybridization. (Additional File 5)

### Branchial Arch enhancer of *pax9*

**Figure 3:**
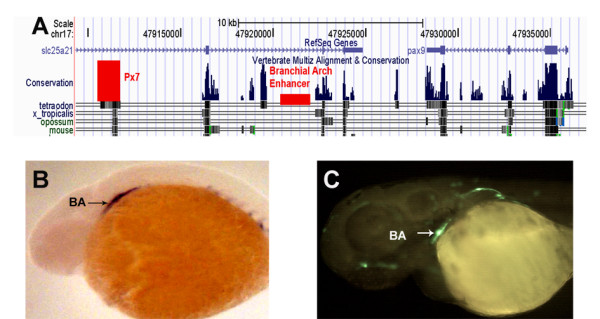
***pax9 *non-conserved enhancer**. (A) UCSC browser snapshot showing synteny around *pax9 *and the location of the functional CNE Px7 and the non conserved enhancer for branchial arch. (B) Whole mount RNA in situ hybridization showing expression of *pax9 *in branchial arch (BA) at 36 hpf. (C) A 500 bp non-conserved sequence drives expression of EGFP in the branchial arch at 36 hpf.

Since *pax9 *expresses in the branchial arch and none of the CNEs around *pax9 *could recapitulate the expression data, we decided to locate the enhancer by testing the whole 15 kb genomic region lying between the last exon of *pax9 *and the functional enhancer Px7. We decided on testing the region on the 3'end of the *pax9 *gene, as the 5' end is gene rich containing *nkx2.9 *and *nkx2.1b *in close proximity. The 3' end only had one gene *slc25a21*, which had introns containing large stretches of non-coding DNA, and also contained one of the enhancer for *pax9 *(Px7). We did overlapping PCRs of about 500 bp each to narrow down the enhancer to a 564 bp region (chr17:47,920,936-47,921,499) (Figure 3A and Additional File 1) that could robustly drive activity in the branchial arch at 36 hpf. (Figure 3B, C). This region does not show sequence conservation even with closely related fish species.

### *pax9 *BAC modification

A 188 kb BAC (CH211-62I6) containing 188 Kb of genomic DNA flanking *Pax9 *was modified with EGFP and injected into 1-cell zebrafish embryo and followed over development. The modified BAC recapitulated the gene expression data in the sclerotome and myotome at 24 to 48 hpf. 70% of the embryos at 48 hpf reproduced this expression pattern in dorsal sclerotome consistently (Figure 2G).

### *otx1b*

**Figure 4:**
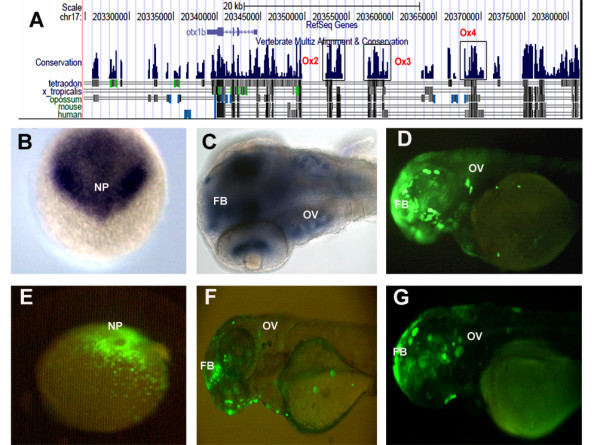
***otx1b *CNES and modified BAC**. (A) UCSC browser snapshot showing synteny around *otx1b *and the functional CNEs Ox2, Ox3 and Ox4. (B) Whole mount RNA in situ hybridization showing expression of *otx1b *in the neural plate at 6 hpf. (C) Whole mount RNA in situ hybridization showing expression of *otx1b *in the forebrain and otic vesicle at 24 hpf. (D) Modified BAC showing expression in forebrain and otic vesicle at 24 hpf. (E) CNE Ox4 drives expression in the neural plate at 6 hpf. CNE Ox2 (F) and Ox3 (G) drives expression in the forebrain at 36 hpf and 48 hpf respectively. Both fail to drive expression in the otic vesicle. FB-forebrain. OV-otic vesicle

*Orthodenticle homolog 1 *is a vertebrate homolog of the *Drosophila orthodenticle *and encodes a member of the bicoid sub-family of homeodomain containing transcription factors. *Otx1 *along with *Otx2 *has been implicated in the regional patterning of the rostral head and both are synergistic in function in certain domains of the brain. The *Otx1 *gene has been strongly associated with the formation of the otic vesicle in gnathostomes starting with the teleost fish and hence helping in transition from the jawless vertebrates to a more gnathostome characteristics [36] and also in determining cerebellar cell identities in zebrafish [37]. Our RNA *in situ *hybridization detected expression of *otx1b *in neural plate at 6 hpf (Figure 4B) and forebrain, midbrain and otic vesicle at 48 hpf (Figure 4C)

### CNEs in and around *otx1b*

Genome alignment revealed 16 CNEs in the syntenic block around *otx1b *(Figure 4A and Additional File 1). All the CNEs were present on the 5' end of the gene with the farthest being 172 kb away. There was no synteny at the 3' end of the gene with mouse and human genomes. Ox4 strongly recapitulated the gene expression at 6 hpf at the neural plate (Figure 4E and Additional File 2). Both Ox2 and Ox3 that are adjacent to each other and about 8-10 kb away from the gene drove EGFP expression in the forebrain at 48 hpf (Figure 4F, G and Additional File 2). We did not detect any EGFP expression from the other CNEs when they were assayed individually with the reporter construct. Thus 19% of the conserved elements assayed in our system were positive as tissue specific enhancers.

### *otx1b *BAC modification

The 111 kb BAC (CH73-220O18) containing the *Otx1 *gene was modified with a reporter and was injected into the 1-cell fertilized zebrafish embryos as previously described. The EGFP expression could be detected as early as 6 hpf in the neural plate (data not shown). An individual CNE also drove reporter gene expression in the same tissue at the same time point and was present in the genomic region cloned in the BAC under testing. 69% of the injected embryos also showed EGFP expression in the forebrain midbrain and otic vesicle at 48 hpf, which overlapped with the endogenous gene expressions at the time point observed by RNA *in situ *(Figure 4D). We detected a much more extensive expression domain for the BAC as compared to the individual CNEs, thus leading us to believe that there exist more regulatory elements in that genomic region than detected by sequence level constrain.

### *foxa2*

**Figure 5:**
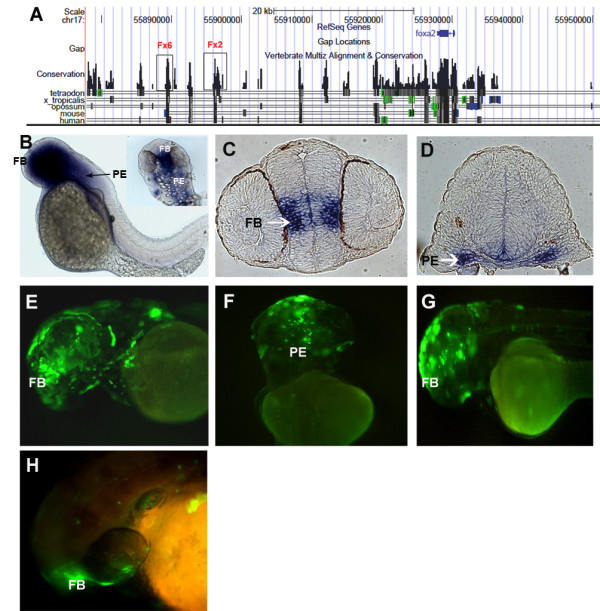
***foxa2 *CNEs and modified BAC**. (A) UCSC browser snapshot showing synteny around *foxa2 *and the functional CNEs Fx2 and Fx6. (B) Whole mount RNA in situ hybridization at 48 hpf shows *foxa2 *expression in the rostral part covering forebrain and pharyngeal endoderm (C) Section in situ hybridization showing expression of *foxa2 *in forebrain at 48 hpf. (D) Section in situ hybridization showing expression of *foxa2 *in pharyngeal endoderm at 48 hpf. (E) Modified BAC showing expression in forebrain and (F) pharyngeal endoderm at 48 hpf.(G) CNE Fx2 and (H) Fx6 drives expression in the forebrain at 48 hpf. FB - forebrain. PE - pharyngeal endoderm

*foxa2 *is a member of the forkhead transcription factor family and is specifically expressed in the visceral endoderm, anterior definitive endoderm, node, notochord and floorplate [38-40]. In zebrafish it has been shown that although *foxa2 *is not required for the induction of the floorplate, it is required for its further differentiation and for induction and/or patterning of several distinct cell types in the ventral CNS [41]. Our RNA *in situ *hybridization detected expression of *foxa2 *along the forebrain and the pharyngeal endoderm at 48 hpf (Figure 5B-D).

### CNEs in and around *foxa2*

Using our criteria, we located six CNEs around *Foxa2*, all of which were located at the 3' end of the gene, the closest being 2 kb away and the farthest being 60 kb away and ranging in size from 425 bp to 1220 bp (Figure 5A and Additional File 1). Each of these CNEs was individually ligated with the reporter construct and the injected embryos were visualized at different time points in development. Two of the CNEs, Fx2 and Fx6 could drive expression in the forebrain at 48 hpf (Figure 5G, H and Additional File 2). There was no detectable expression of EGFP from the other individual CNEs at the time points under observation. So only 33% of the CNEs assayed were functional as developmental enhancers in zebrafish.

### *foxa2 *BAC modification

The 179 kb BAC (CH211-38M14) containing the genomic insert in and around zebrafish *foxa2 *was modified and injected into 1-cell zebrafish embryos. The BAC recapitulated the endogenous gene expression particularly in the rostral end of the embryo in the forebrain and pharyngeal endoderm at 48 hpf (Figure 5E-F). We failed to detect EGFP expression in any other *foxa2 *expression domains.

### Bac dissection to detect non-conserved enhancers

**Figure 6:**
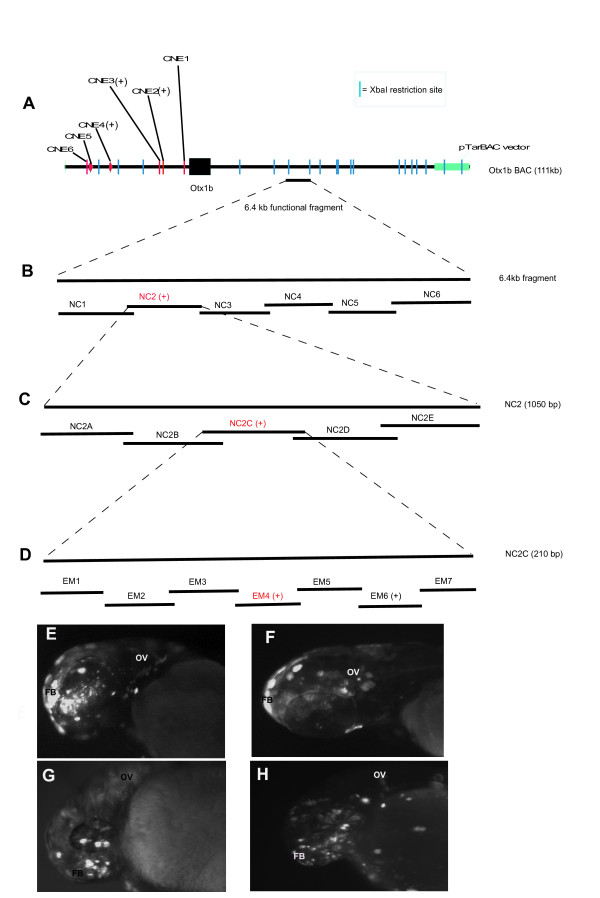
**BAC dissection to detect *otx1b *non-conserved enhancer**. (A) The *Otx1b *111 kb BAC showing the gene and the 6.4 kb non-conserved functional element and the CNEs present. The blue lines indicate the XbaI restriction sites. The plus sign in parenthesis indicates functional CNEs. (B) Six overlapping PCR fragments covering the 6.4 kb fragment. (C) Five overlapping PCR fragments covering the 1050 NC2 functional fragment (D) the seven Cy5 labeled probes designed on the functional NC2C fragment. Transgenic assay with the whole BAC (E), 6.4 kb fragment (F), NC2 (1050 bp) fragment (G) and NC2C (210 bp) fragment (H) all at 24 hpf.

Since many of the CNEs failed to act as enhancers coupled with the fact that our data on *pax9 *as well as previous studies reported the presence of non-conserved enhancers [20] we sought to investigate in a detailed manner if we could locate more such functional non-conserved enhancers. Since most of our modified BACs gave us expressions that were more extensive than the individual CNEs alone, we employed the traditional method of BAC dissection. We selected the *otx1b *BAC for these experiments as the modified BAC for this gene had shown a robust spatio-temporal expression, recapitulating substantial domains of expression of the endogenous gene (Figure 6E). The BAC was digested with the restriction enzyme XbaI, which generated 22 fragments ranging from 48 bp to 27 kb in size (Figure 6A). The fragments were individually ligated to the minimal reporter construct and injected into 1-cell zebrafish embryo. Three fragments gave EGFP expression in the fore and/or mid brain of the zebrafish embryo at 24 and 48 hours. Two of the positive fragments each contained a functional CNE in them (Ox2 and Ox3). The third fragment, a 6.4 kb piece from the 3' end (chr17:20,307,773-20,314,262) of zebrafish *Otx1b *gene mapped to a large region that included introns and exons of a predicted gene (Ensembl Transcript: ENSDART00000090374). Also this fragment was conserved in among the fishes-Fugu and Tetraodon. This element was functional in our assay expressing in forebrain and otic vesicle (Figure 6F). The fact that it mapped to such a large region that included exons led us to believe that the core element might be smaller. This 6.4 kb (NC) regulatory element was further reduced into five overlapping 1050 bp and one 1500 bp fragment by PCR (Figure 6B). The overlapping PCR fragments ensured we did not have abrupt breakpoints in the DNA fragments. These six fragments (NC1-NC6) were individually tested by co-injection with the minimal reporter construct into zebrafish embryos and one (NC2) of the 1050 bp fragments (chr17:20,312,333-20,313,342) partially recapitulated the expression domain of the bigger 6.4 kb fragment at 24 and 48 hpf (Figure 6G). There was no expression from the other 5 fragments. NC2 showed partial conservation to the tetraodon and fugu genome. NC2 was further reduced to five (NC2A-NC2E) 210 bp fragments by doing overlapping PCRs and each piece was individually injected with a reporter construct (Figure 6C). Fragment NC2C (chr17:20,312,743-20,312,952) on its own could drive expression of EGFP reporter in a subset of the expression domain of NC2 (Figure 6H), which was a subset of the expression domain of the 6.4 kb fragment in the forebrain and otic vesicle. Interestingly the NC2C 210 bp fragment had no conservation with any species in the genome alignment. Thus we concluded that this element was a zebrafish specific enhancer for *otx1b*. We also observed that reducing the size of the DNA resulted in a slight reduction both in the domain of expression as well as the level of expression in the embryo. One possible reason for this reduction in expression domain was that, although the small 210 bp fragment contained most of the transcription factor binding sites essential for its function, it was missing some other binding sites which were in the neighboring fragments and the extensive expression domain as seen by the 6.4 kb fragment is result of the synergistic activity of multiple transcription factors interacting with the regulatory element.

### Biochemical and transgenic assays to detect a core binding site

**Figure 7:**
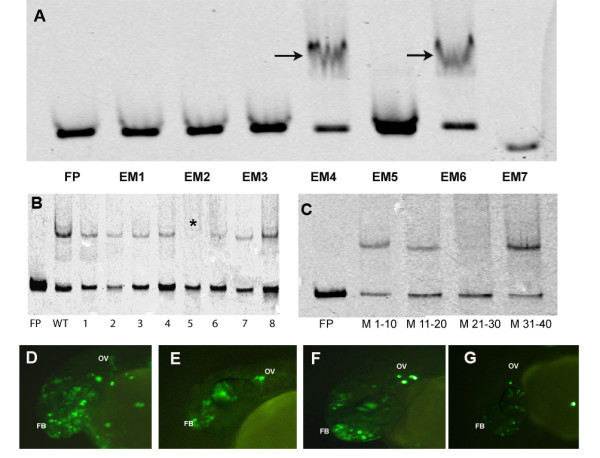
**EMSAs and transgenic assays to detect core-binding motif**. (A) Electrophoretic Mobility Shift Assays (EMSA) with 7 overlapping probes spanning the 200 bp non-conserved enhancer. Arrows indicate the two probes that bound protein. (B) EMSAs with 5 bp sliding mutant probes for EM4. The star indicates the probe 5 which did not show binding and the subsequent probe 6 showed weak binding. (C) EMSA with 10 bp mutation probes. Mutations in nucleotides 21-30 (M21-30) leads to complete abrogation of binding. Transgenic assays with wild type 40 bp probe (EM4) (D), mutant probe 5 (E), mutant probe 6 (F) and 10 bp mutant probe (G). The enhancer could drive expression in forebrain (FB) and otic vesicle (OV) at 24 hpf that are domains of expression of *otx1b*.

Seven overlapping Cy5 labeled DNA probes were designed to span the 210 bp *Otx1b *non-conserved enhancer (NC2C) (Figure 6D). The probes were incubated with nuclear extract from 24 hpf zebrafish embryos and run on a native gel (EMSAs). Two out of the seven fragments showed a distinct shift indicating that they contained sites for transcription factor binding (Figure 7A). Both of these fragments were co-injected with the EGFP reporter construct into 1-cell zebrafish embryos. One of the fragments (EM4) could drive EGFP expression to the rostral portion of the brain, recapitulating a subset of the expression domain of the bigger 200 bp fragment (Figure 7D). The other fragment (EM6) that bound protein in vitro did not show any activity in vivo.

We made "sliding" 5 bp mutations (nucleotide transversions e.g. A to C) along the 40 bp (EM4) region to narrow down the binding site (Additional File 6). Mutations in nucleotide 21 to 25 (GGAGG) completely abrogated binding of the protein with weakened binding observed for mutations in nucleotides 26-30(GAGGG) (Figure 7B). Injection of the EGFP reporter construct harboring the mutant 5 bp binding sites independently in the zebrafish showed weakened EGFP expression but not complete loss of activity (Figure 7E, F). This led us to believe that the nucleotide 21-30 together harbored the functional site in the enhancer. We went ahead and designed mutant probes for the whole 10 bp (21-30 nucleotides) region and EMSA and transgenic assays showed a complete abrogation of binding and markedly reduced EGFP expression in the zebrafish embryos (Figure 7C, G). This gave conclusive proof that the actual binding site and the core functional domain of the non-conserved enhancer lay within these 10 nucleotides.

### Enhancer binding transcription factor

**Figure 8:**
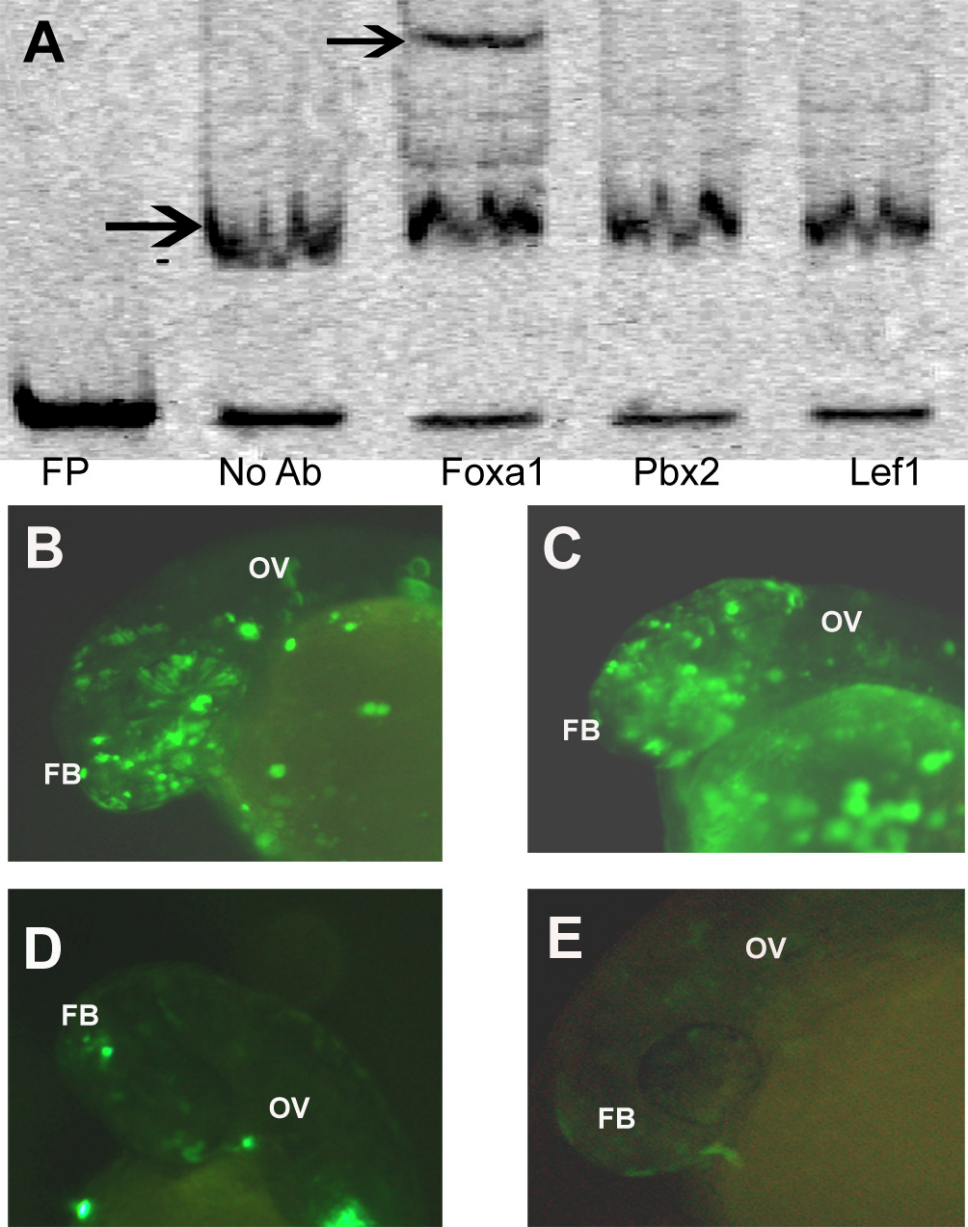
**Elucidation of transcription factor binding to the enhancer**. (A) Supershift assay with Foxa1, Pbx2 and Lef1 antibodies. Black arrow in no Ab lane denotes the shift and the one in Foxa1 added lane denotes supershift. (B) Expression of the 40 bp sequence (EM4) driven EGFP construct in forebrain (FB) and otic vesicle (OV) which are domains of expression of *otx1b *at 24 hpf. (C) Co-injection of the 40 bp enhancer along with a scrambled morpholino, showing no down regulation of the enhancer activity at 24 hpf. (D) Co-injection of the 40 bp enhancer along with morpholino 1 (M1) against *Foxa1 *showing reduction in the enhancer activity at 24 hpf. (E) Co-injection of the 40 bp enhancer along with morpholino 2 (M2) against *Foxa1 *showing reduction in the enhancer activity at 24 hpf.

We used the TRANSFAC database (http://www.gene-regulation.com/index.html) [42] to find potential transcription factors (TFs) that could bind to the 10 bp sequence in our non-conserved enhancer. By using a criterion of entire matrix similarity > 0.75 we came up with a list of 3 putative transcription factors that could bind the sequence: Foxa1, Pbx2, and Lef1 (Additional File 6). We performed supershift assays with antibodies against the potential TFs and saw a supershift uniquely with the Foxa1 antibody (Figure 8A). To conclusively prove that Foxa1 was critical for the functioning of the enhancer we designed 2 morpholinos against Foxa1 and co-injected the morpholinos with the 40 bp non-conserved enhancer construct. Whereas the scrambled morpholino (Figure 8C) did not have any effect on the enhancer and recapitulated the expression of the enhancer alone (Figure 8B), knocking down Foxa1 resulted in a dramatic reduction of the enhancer activity by both the morpholinos (Figure 8D, E). To verify if Foxa1 was indeed knocked down in the morphants, we did western blots, as previously described [34], to detect proteins levels of Foxa1. The western blots indicated that both the morpholinos used against Foxa1 resulted in dramatic decrease in levels of Foxa1, which were not seen in the scrambled morpholino control (Additional File 7 Figure A). To test if downregulation of Foxa1 and decreased activity of the *otx1b *enhancer actually led to a dramatic drop in expression of *otx1b *we did RT-PCR on RNA extracted from both wild type as well as morphant embryos at 24 hpf (Additional File 7 Figure B) as well as RNA *in situ *for *otx1b *in both these embryos (Additional File 7 Figure C). There was no discernable expression change detected by PCR or *in situ*, strongly suggesting that the expression of *otx1b *at 24 hpf is also controlled by other functional enhancers thus the abrogation of activity of one regulatory element does not lead to significant drop in its expression. Hence our data suggests that Foxa1 is important in driving the activity of *otx1b *in the forebrain and otic vesicle in zebrafish via a redundant enhancer which is not conserved at the sequence level.

## Discussion

This study focused on examining CNEs directing expression of four developmental control genes that are ancient in origin and conserved during evolution and expressed in the three different germ layers. This allowed us to ask two very important questions regarding the nature and role of these conserved elements: (1) are all the conserved elements found in the syntenic region around a gene functional? (2) Can we find all the enhancers for a particular gene by looking only at the CNEs? The CNEs selected for our study were selected based on a criteria of ≥ 60% sequence identity and ≥ 100 bp in length across three phylogenetically distant species (human, mouse, zebrafish). As the teleost fishes and mammals last shared a common ancestor about 450 million years ago, predating the emergence of the majority of all extant vertebrates, it strongly implied that any non-coding sequences conserved between these two groups are likely to be fundamental to vertebrate life and hence functional. The design of our study has also allowed us to address some major arguments which have been put forward before to explain the absence of functionality in these CNEs in human-mouse comparative studies. By being able to look at potentially all developmental time points we had an advantage over most of the studies where the functional testing was done in mouse and was restricted to one developmental time point [14]. Yet our data clearly indicates that even DNA elements that are conserved very stringently over long evolutionary time might not have functional roles as enhancers during development. The fact that many of these conserved non-coding elements are non-functional as enhancers and non-conserved DNA can be functional is not totally unexpected. Previous studies [9,20] by other groups looking at single genes also revealed similar trends in other genomic loci in the zebrafish. Conversely it has also been shown that absence of sequence level homology does not preclude a fragment of DNA from being functional as a developmental enhancer [13,43]. Thus our data supports the hypothesis that in zebrafish many of the enhancers for important developmental control genes are not constrained at the sequence level.

We have also demonstrated that there is still a need to combine high throughput comparative genomics with detailed biochemical assays to locate and validate *cis-*regulatory elements in the vertebrate genome. By doing such detailed assays on one of the non-conserved enhancers we have demonstrated that putative transcription factor binding sites and the actual transcription factor binding to an enhancer can be determined with great accuracy and can lead to a more complete analysis of an enhancer. Though a recent study discovered motifs in zebrafish enhancers, they only predicted what putative transcription factors could bind them [44], we went beyond only prediction and actually validated the responsible transcription factor. We believe our study will help in opening the door for future studies looking in finer detail at individual enhancers and how they control gene expression.

We have also shown the utility and efficiency of using BAC transgenics in zebrafish to locate and validate distal *cis*-regulatory elements for different genes. BACs have been successfully used to validate *cis*-regulatory elements for numerous genes in mouse [28,45] and in certain cases multiple overlapping BACs have been used to locate all the regulatory elements of a gene [27]. BAC modification for transgenics has also been employed for a few zebrafish genes in the past [46-49]. We have done an extensive spatio-temporal gene expression study of BAC transgenics for four critical developmental control genes. Thus we have demonstrated that circular modified BACs can be used without linearization for large-scale transgenic studies in zebrafish making it an attractive model system for similar future studies.

Finally it has not escaped our attention that only about 20-30% of the CNEs tested were functional as developmental enhancers. Most of these fragments could indeed be non-functional but alternately other possible explanations exist. One possibility is that these elements are acting in a synergistic manner and require other elements together to function. This was also demonstrated by our BAC studies, which show much more extensive expression patterns as compared to the individual CNEs. Also the possibility exists that these CNEs could be negative regulatory elements and hence our assay fails to capture this activity. Some recent results from other groups have indicated the same for the human genome [15]. Also other functions such as chromatin attachment sites, miRNA genes or splice regulatory regions may also reside in such highly conserved non-coding sequences [50-54].

## Conclusions

Thus it is becoming increasing clear that the degree of sequence conservation is not a measure of how 'important' or 'crucial' a functional element might be, but its relevance is shaped by the underlying molecular mechanisms that determine its particular function. Hence as we demonstrated here, future needs will be to combine evolutionary, functional and bioinformatic approaches to understand how these sequences function at the molecular level to determine the nature of these interactions and finally decipher what 98% of our genome encodes.

## Methods

### RNA in situ hybridization

DNA clones for making *in situ *hybridization probes were obtained from the Expressed Sequence Tag (EST) clone collection at the Genome Institute of Singapore and whole mount *in situ *hybridization using digoxigenin (Roche) labeled RNA probes was performed as described previously [55]

### Fish maintenance and embryo staging

Fish were maintained in the GIS zebrafish facility according to standard procedures. Crosses were set up in the evening and the barrier was lifted the next morning. After half an hour, the fertilized embryos were collected and maintained at 28.5°C in egg water supplemented with methylene blue. The stages of embryos were indicated as hpf (hours post-fertilization) or dpf (days post-fertilization) [56]. From 24 hours onward the embryos were raised in egg water containing Phenylthiourea. This helps to prevent pigmentation and allows better visualization of the Enhanced Green Fluorescent Protein (EGFP) signal. The GFP2 filter of stereomicroscope (Leica Microsystems, Germany) was used to observe the EGFP signals in transgenic zebrafish. The Leica DFC camera was used to take photographs and was captured using the IM50 software from Leica.

### Genome alignments and Conserved Non-coding Elements (CNEs)

The University of California Santa Cruz (UCSC) genome browser's (http://genome.ucsc.edu) zebrafish (*Danio rerio*) Zv6 genome assembly was used as a base genome to align the genome sequences with mouse (mm8 assembly) and human (hg18 assembly) for regions around genes of interest. This assembly was used initially as the comparative genomics tracks were best annotated. Aligning the sequences with a recent assembly (Zv8) has shown that all the CNEs tested have still remained conserved and non-coding in the new annotation. For the positive CNEs the new coordinates from the Zv8 assembly are listed in Additional File 1. The closest gene to the start of each CNE was determined by the looking at known gene annotations on the UCSC genome browser. The UCSC browser's conservation tracks (phastcon conserved elements) were used to determine conserved elements (≥ 60% identity, ≥ 100 bp length). Transcribed sequences in the conserved set were filtered out using known genes, spliced ESTs and mRNA annotations (intronic conservation was allowed). We confined our search for CNEs up to 1 Mb or till wherever there was a break in synteny around the gene of interest between the three species (zebrafish-mouse-human).

### Functional Assays

We assayed for enhancer activity in embryos by either co-injecting the candidate enhancer element with a minimal promoter-reporter vector in a method previously described [19,57] or by ligating individual CNEs to the minimal vector. Though there has been suggestions that using transposon based vectors might be a more efficient way to assay for enhancers, our previous study and also studies from other labs have shown that circular vectors containing the enhancer-reporter construct as well as co-injection of the reporter construct with the DNA are equally efficient in transient transgenic assays in zebrafish [34,58]. In a series of control experiments we injected just the reporter construct alone (negative control), which was found to be transcriptionally inactive on its own. For preparation of DNA for microinjections, CNEs were PCR amplified from zebrafish genomic DNA or bacterial artificial chromosomes (BACs) containing the region of interest (50-100 bp sequences immediately flanking the core conserved region on either side was included in the PCR). DNA was purified using the QIAquick PCR purification kit (Qiagen, USA). The minimal vector was constructed by cloning in the EGFP ORF (Clontech, USA) under the control of a minimal promoter from the mouse β-globin gene. This fragment was released from the vector backbone using SmaI restriction enzyme and gel purified using QIAquick gel extraction kit (Qiagen, USA). Element DNA (at 20-50 pg/μl), reporter fragment (50 pg/μl) and phenol red (0.1% as tracer) were combined and injected into at least 200 1-cell zebrafish embryos using a Pico-Injector (PLI-100 Harvard Instrument, USA). Three independent injections were done for all constructs (Additional File 2).

For locating the *pax9 *non-conserved branchial arch enhancer, we did 500 bp overlapping PCRs spanning the 15 kb genomic region (chr17:47,911,703-47,927,438) from the final exon of *pax9 *to the functional CNE Px7 which is located in the third intron of the neighboring gene *slc25a21*. Each fragment was ligated to the reporter construct individually tested. We did not PCR out the three exons of the *slc25a21*. The embryos were observed at various time points in development (from 6 hpf to 3 dfp) for GFP expression. On basis of the GFP expression a CNE was scored as 1) positive, 2) negative or 3) positive but not in "expected" domain of the gene. The domains of expression were annotated by referring to the RNA *in situ *hybridization results for the gene and also by cross-referring to existing gene expression data at ZFIN (The Zebrafish Model Organism Database) (http://www.zfin.org).

### BAC targeting construct and modification

The UCSC genome browser was used to locate 150-250 kb BACs containing the gene of interest. A BAC was selected which contained sufficient flanking genomic sequences around the gene. This enabled us to retain the regulatory elements in their proper genomic context and hence increase the possibility of capturing the maximum number of distal *cis-*regulatory elements. A BAC targeting vector was constructed by PCR amplifying a FRT-PGK-gb2-NEO-FRT cassette from a commercially available vector (Gene Bridges, GmbH). This was cloned into the NotI site of a pBlueScript vector SK+ (Stratagene, USA) containing the EGFP ORF between its XhoI-NotI sites. This fragment containing the EGFP ORF and selection cassette was PCR amplified with 50 bp homology arms on either side of the translational start site of each gene of interest and homologously recombined using the GeneBridges BAC modification kit (Gene Bridges, GmbH) as per the manufacturer's instructions (Additional File 4) [59]. Circular modified BAC DNA was purified using Qiagen Maxiprep kit and injected into one-cell zebrafish embryo and EGFP expression followed over development.

### BAC dissection

The *otx1b *BAC was digested with XbaI restriction enzyme and the fragments generated were co-injected with the reporter vector into embryos. The fragments that drove EGFP expression were mapped back to the zebrafish genome assembly to determine its position with respect to the *otx1b *gene. The 6.4 kb non-conserved fragment was further subdivided into 1 kb and 200 bp fragments by generating overlapping PCRs. The 200 bp PCR generated genomic fragment that drove expression of EGFP was used in subsequent EMSAs.

### Electrophoretic mobility shift assays (EMSAs)

Nuclear proteins were extracted from 24 hpf zebrafish embryos using the NE-PER nuclear and cytoplasmic extraction kit (Thermo Scientific, USA). EMSAs were carried out using DNA probes modified with 5' Cy5 labels (Sigma Proligo). The 200 bp non-conserved functional element was sub divided into six 40 bp and one 30 bp overlapping fragments having a 10 bp overlap. Equimolar amounts of complementary strands were mixed and heated to 95°C followed by gradual cooling to ambient temperature over at least 5 h to anneal the probes. For binding studies, double-stranded DNA probes at 10 nM were mixed with 10 ug of nuclear proteins and 500 ng of Poly dI-dC (Sigma) in a buffer containing 40 mM Tris-HCl (pH 8.0), 0.4 mg/ml BSA, 200 uM ZnCl_2 _, 400 mM KCl, 40% glycerol and 0.4% IGEPAL and incubated at 4°C in the dark for one hour. The bound and unbound probes were subsequently run on a pre-run 8% 1X TBE polyacrylamide gel for approximately 30 min at 200 V. The fluorescence was detected using a Typhoon 9140 PhosphorImager (Amersham Biosciences). At least three independent experiments for each binding site were performed to ascertain binding.

Mutation studies on the 40 bp genomic DNA fragment (EM4) that showed a shift was carried out by sequentially mutating 5 and 10 bases each along the sequence by transversion and carrying out EMSA on each mutant probe. For supershift experiments, 10 ug of 24 hpf zebrafish nuclear extract was incubated with 10 nM EM4 probe and 1 ug of antibody against the three predicted transcription factors (FOXA1: ab23738, PBX2: ab 66942, LEF1: ab 52017, all from Abcam plc), incubated for 1 hour and run on a gel as described above.

### Morpholinos

The following two morpholinos against *foxa1 *were injected into about 200 1 to 2-cell embryos at a concentration of 0.6-1.2 pico molar: 5'-CGCCCAACATTATGGAGGAAATCC-3' (M1) and 5'-CTTCCATTTTCACTGCGCCCAACAT-3' (M2). The specificity of *foxa1 *morpholinos was confirmed by using a standard scrambled control morpholino (CMO) 5'CCTCTTACCTCAGTTACAATTTATA-3' (Gene Tools).

### Western Blot

Western blot was carried out as previously described [34] with some modifications. 10 morphants and wild type 24 hpf zebrafish was used to extract protein using the NE-PER nuclear and cytoplasmic extraction kit (Thermo Scientific, USA) and 25 ug of nuclear extract was loaded on the gel. Foxa1 antibody (ab23738, Abcam Plc) was applied in 1:500 dilution, followed by anti mouse IgG at 1:2500 dilution.

### RT-PCR

Total RNA was extracted from wild type and morphant 24 hpf zebrafish using TRIzol (Invitrogen, USA) as per manufacture's instruction. Reverse transcription was performed using Superscript III (Invitogen, USA) and oligo DT primers. PCR was performed on the cDNA using the primers 5'-AATCTCCATCCGTCTACATT-3' and 5'-CAGGCCGTTCATGGCGTAGG-3' for *otx1b *and 5'-CGGTGACATCAAGGAGCT-3' and 5'-TCGTGGATACCGCAAGATTCC-3' for *b-actin*.

## Authors' contributions

SC participated in the design of the study, carried out the experimentation, helped in the interpretation of the results and help to draft the manuscript. GB participated in the interpretation of the data. TL conceived of the study, and participated in its design and coordination and helped to draft the manuscript. All authors read and approved the final manuscript.

## Supplementary Material

Additional file 1 **Genomic Location of CNEs tested**. Genomic locations of all CNEs tested and details of PCR primers. Click here for file

Additional file 2 **Zebrafish transient transgenics**. Numerical details of the transient transgenic experiments in zebrafish. Click here for file

Additional file 3 **Immunohistochemistry for EGFP driven by *nkx3.2 *enhancer**. (A) Enhancer Bx4 driven EGFP expresses in the sclerotomal cells (black arrow) at 24 hpf. As marked by *nkx3.2 *in the section RNA *in situ *hybridization (B) Click here for file

Additional file 4 **BAC modification process**. (A) The UCSC genome browser showing two BACs (red arrows) spanning the gene *nkx3.2 *in zebrafish. (B) BAC modification by homologous recombination to insert a reporter gene and a drug selection cassette next to the translation start site of the gene (ATG). (C) A zebrafish carrying the modified BAC for the gene *nkx3.2 *expressing EGFP in sclerotomes (white arrows) at 24 hpf. Abbreviations: EGFP, enhanced green fluorescent protein; NEO, neomycin; LHA, left homology arm; RHA, right homology arm; TGA, stop codon. Click here for file

Additional file 5 **Immunohistochemistry for EGFP driven by *pax9 *enhancers**. (A) Enhancer Px2 driven EGFP expresses in the sclerotomal cells (black arrow) at 36 hpf. The red arrow indicates background staining. Similar expression domains can be seen for enhancer Px4 (B) at 36 hpf and Px7 (C) at 48 hpf. (D) the negative control (empty vector injection) shows no staining in the sclerotome. (E) Section RNA *in situ *hybridization for *pax9 *at 36 hpf. Click here for file

Additional file 6 **Probe sequences used for EMSA and TRANSFAC details**. Mutants probes used for EMSA and TRANSFAC score for putative transcription factors bound to the enhancer. Click here for file

Additional file 7 **Western Blot and RT_PCR in Foxa1 morphants**. (A) Whole zebrafish nuclear extracts probed with Foxa1 antibody. Wild type (WT), Scrambled morpholino (CMO). Morpholino 1 (M1), Morpholino 2 (M2). LOWER PANEL: anti Histone H3 blot to show loading control. (B) RT-PCR on RNA extracted from 24 hpf zebrafish embryos. Lane A: WT, Lane B: M1, Lane C: M2. (All *otx1b *primers). Lane D-F. PCR on same samples using *b-actin *primers as internal control. M-100 bp ladder. (C) RNA *in situ *hybridization for *otx1b *in WT and Foxa1 morphant embryos. Click here for file
