# Supplementary figures and images for: Conserved and non-conserved enhancers direct tissue specific transcription in ancient germ layer specific developmental control genes
Source: BMC Dev Biol. 2011 Oct 20;11:63. doi: 10.1186/1471-213X-11-63 (PMC3210094; doi:10.1186/1471-213X-11-63)

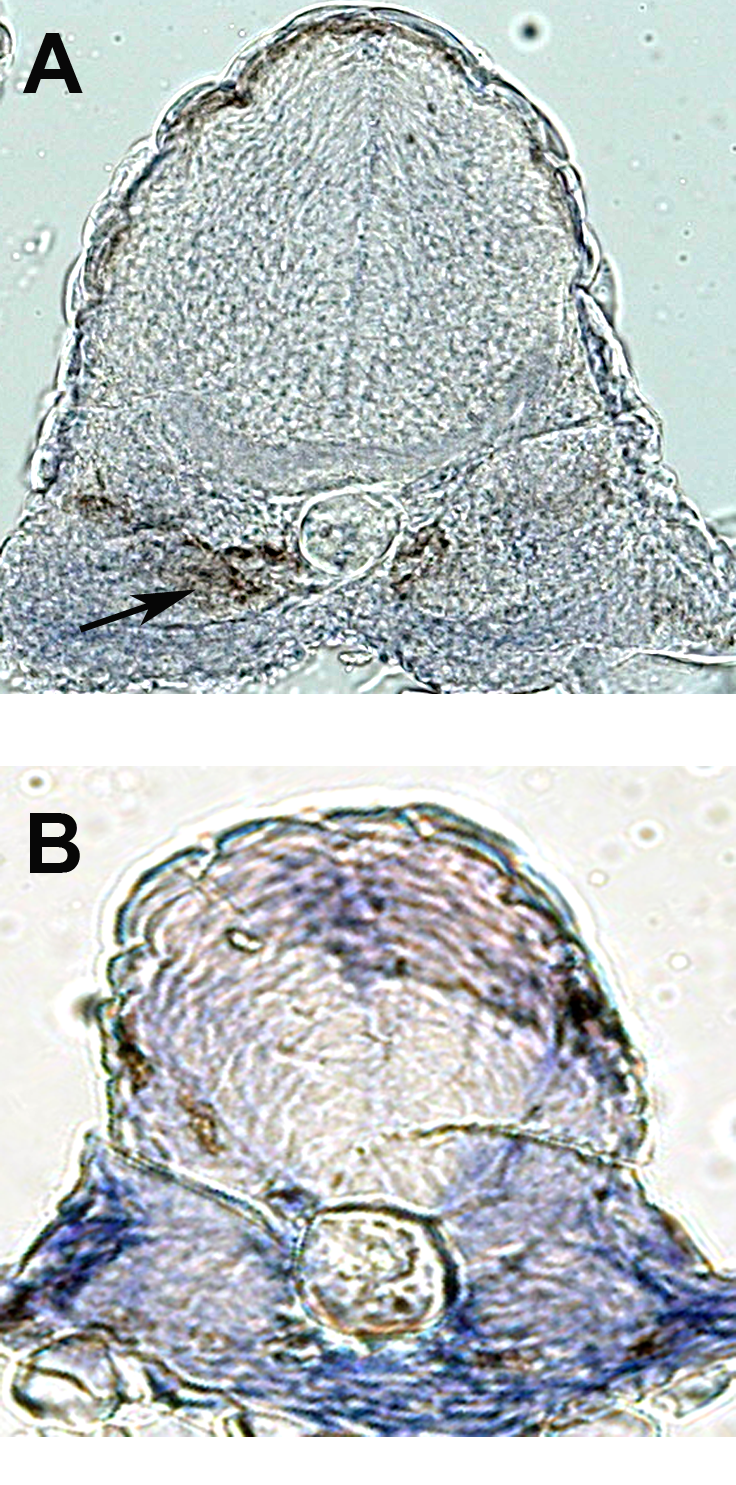

Supplement: Additional file 3 — Immunohistochemistry for EGFP driven by nkx3.2 enhancer. (A) Enhancer Bx4 driven EGFP expresses in the sclerotomal cells (black arrow) at 24 hpf. As marked by nkx3.2 in the section RNA in situ hybridization (B) [file 1471-213X-11-63-S3.TIFF]

**A**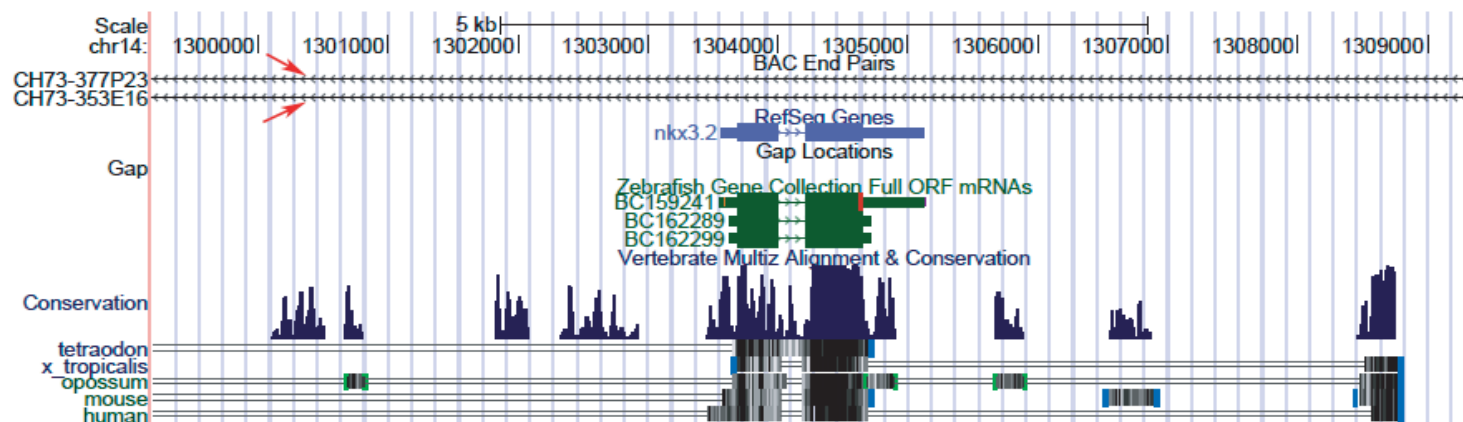**B**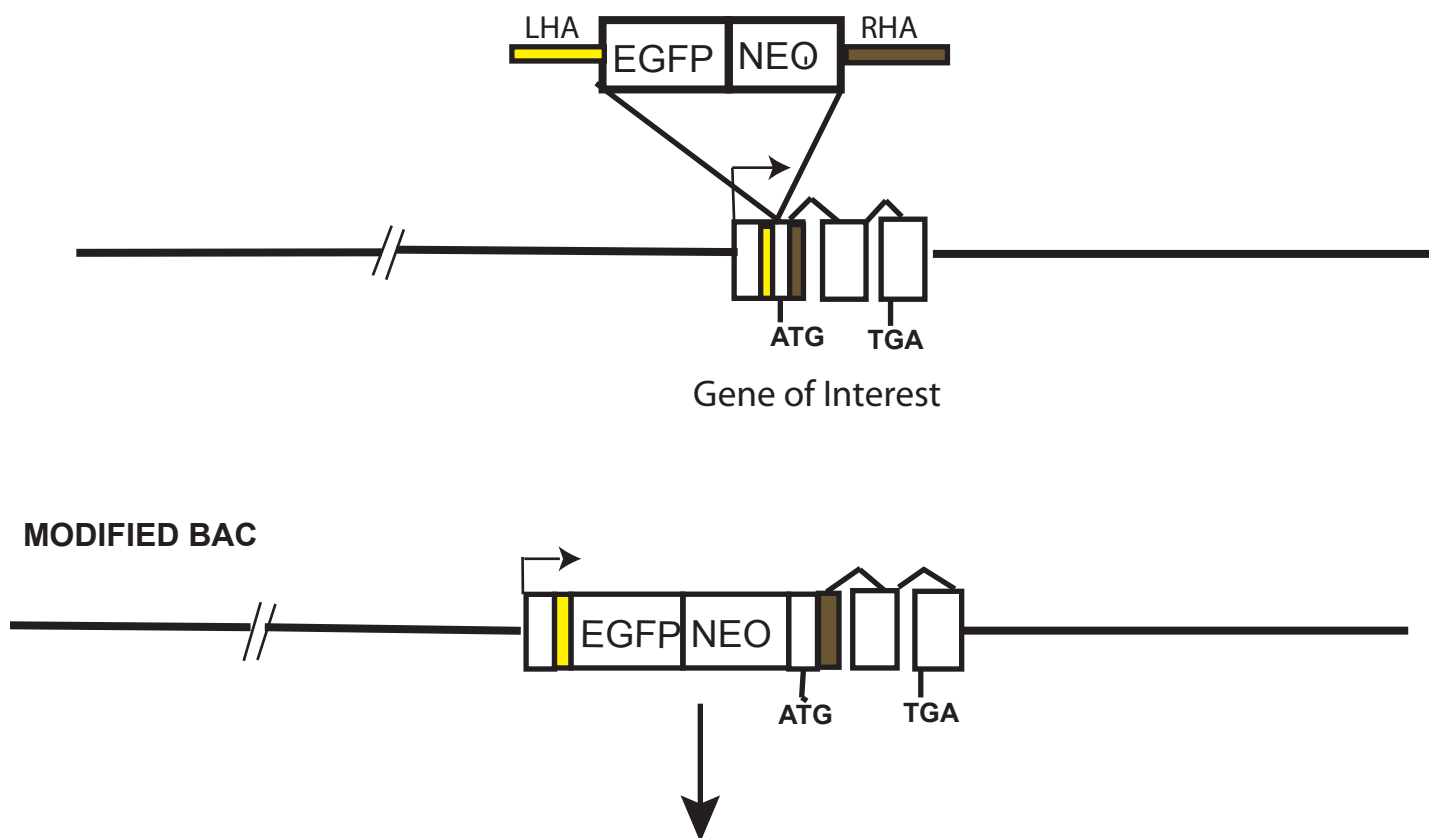**C**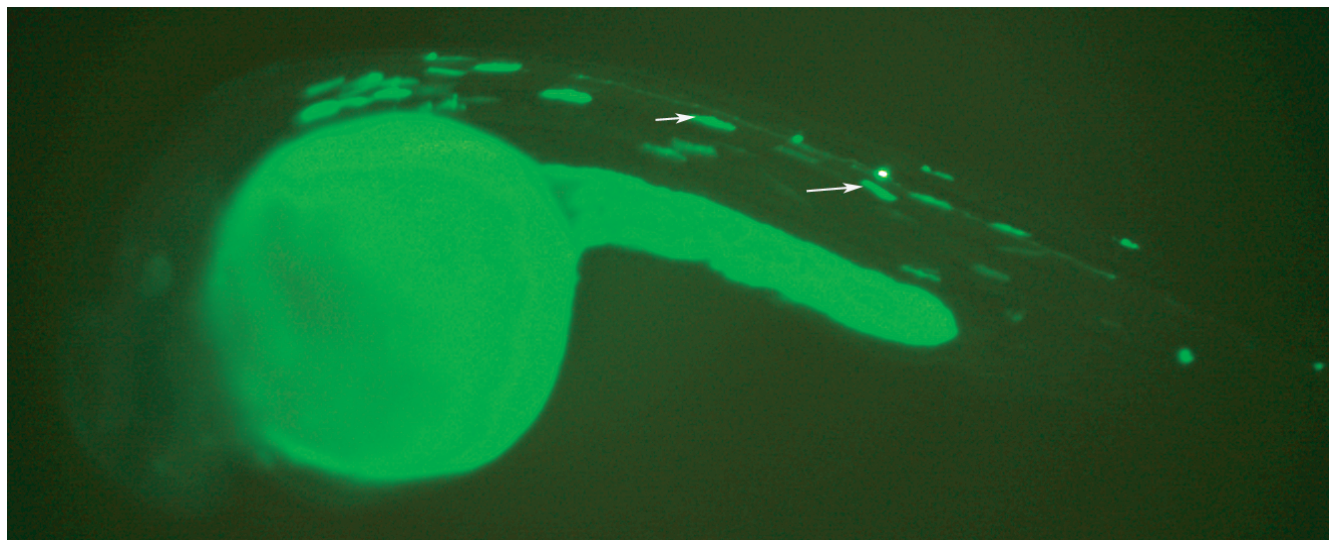

Supplement: Additional file 4 — BAC modification process. (A) The UCSC genome browser showing two BACs (red arrows) spanning the gene nkx3.2 in zebrafish. (B) BAC modification by homologous recombination to insert a reporter gene and a drug selection cassette next to the translation start site of the gene (ATG). (C) A zebrafish carrying the modified BAC for the gene nkx3.2 expressing EGFP in sclerotomes (white arrows) at 24 hpf. Abbreviations: EGFP, enhanced green fluorescent protein; NEO, neomycin; LHA, left homology arm; RHA, right homology arm; TGA, stop codon. [file 1471-213X-11-63-S4.PDF]

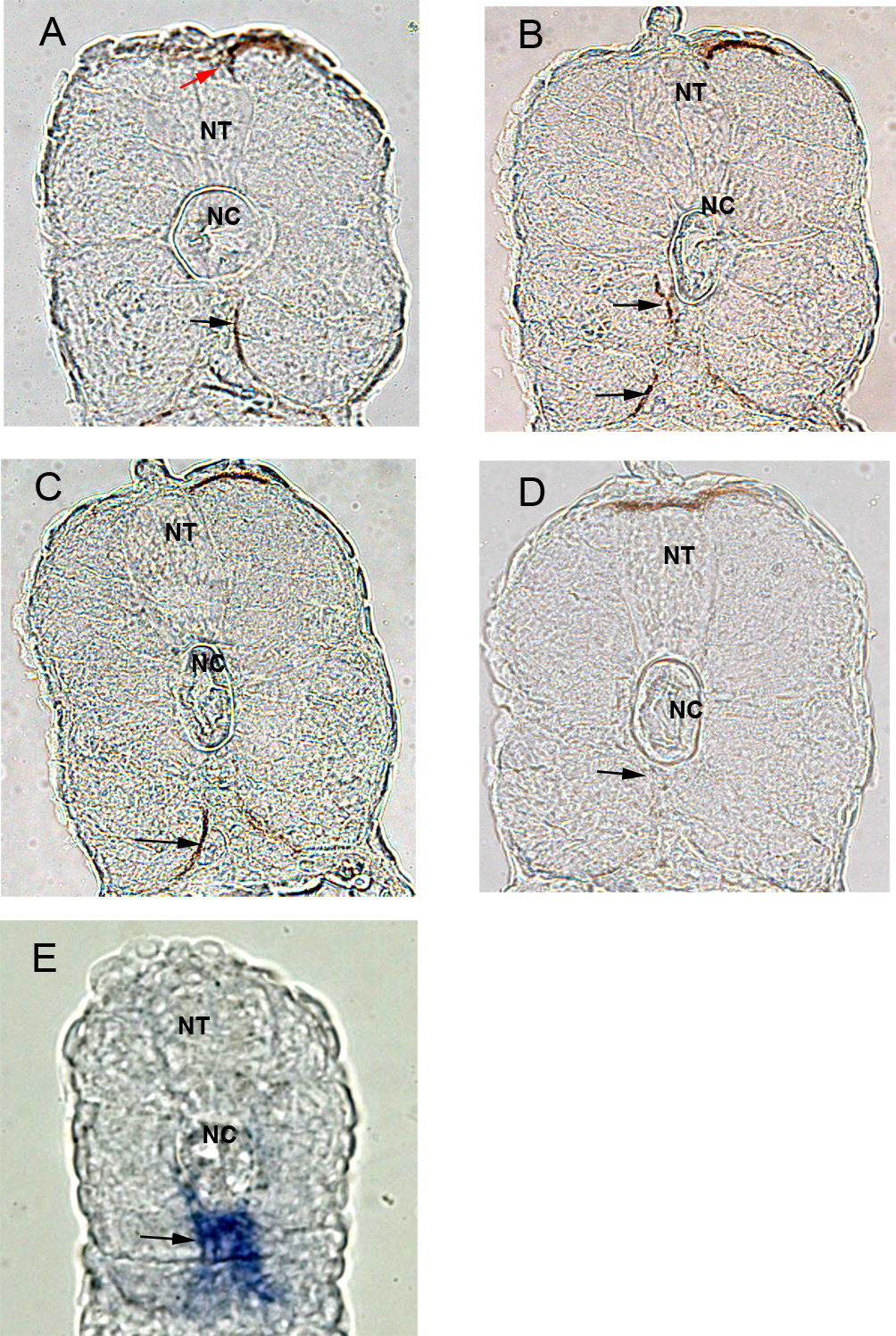

Supplement: Additional file 5 — Immunohistochemistry for EGFP driven by pax9 enhancers. (A) Enhancer Px2 driven EGFP expresses in the sclerotomal cells (black arrow) at 36 hpf. The red arrow indicates background staining. Similar expression domains can be seen for enhancer Px4 (B) at 36 hpf and Px7 (C) at 48 hpf. (D) the negative control (empty vector injection) shows no staining in the sclerotome. (E) Section RNA in situ hybridization for pax9 at 36 hpf. [file 1471-213X-11-63-S5.TIFF]

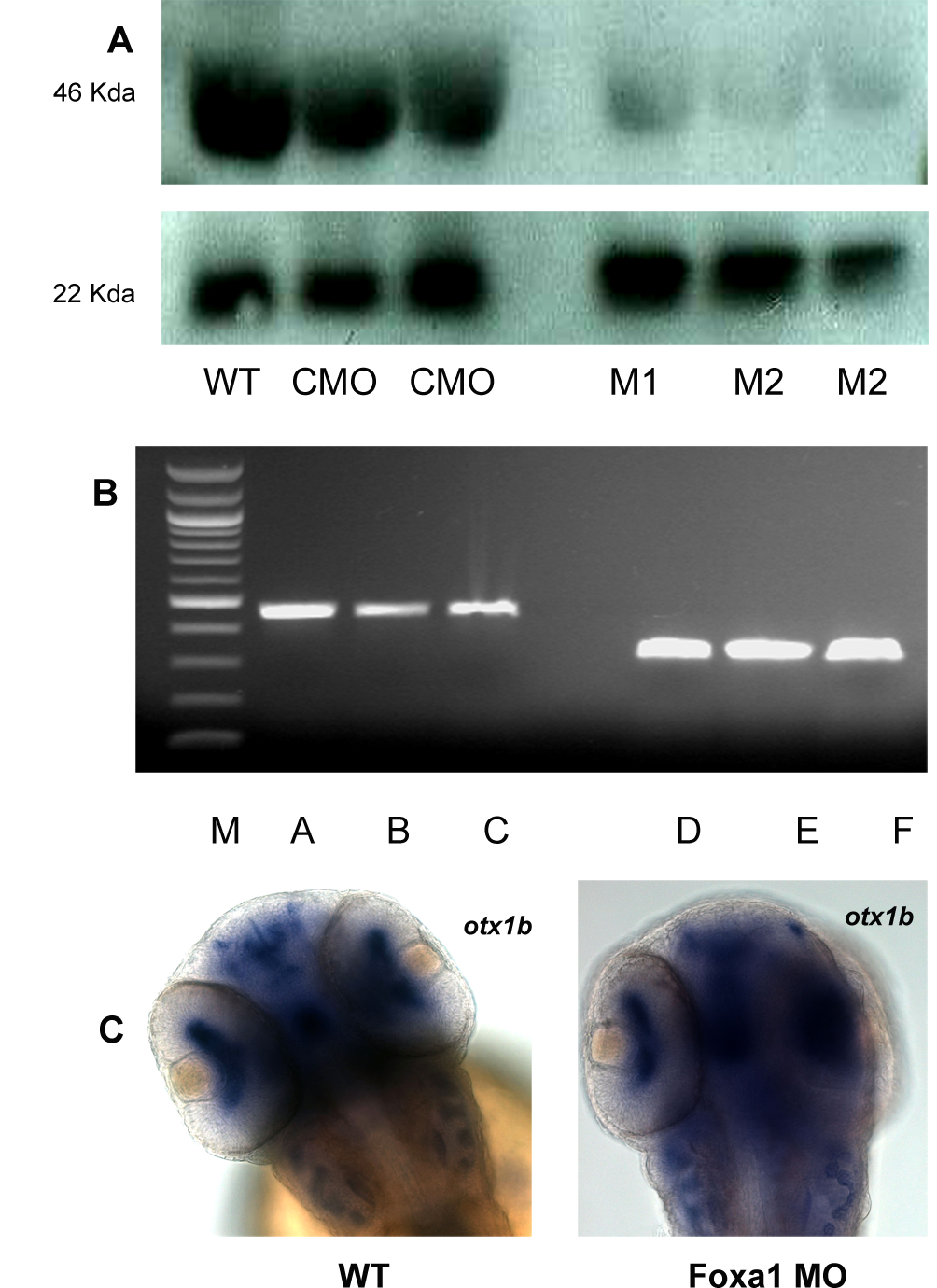

Supplement: Additional file 7 — Western Blot and RT_PCR in Foxa1 morphants. (A) Whole zebrafish nuclear extracts probed with Foxa1 antibody. Wild type (WT), Scrambled morpholino (CMO). Morpholino 1 (M1), Morpholino 2 (M2). LOWER PANEL: anti Histone H3 blot to show loading control. (B) RT-PCR on RNA extracted from 24 hpf zebrafish embryos. Lane A: WT, Lane B: M1, Lane C: M2. (All otx1b primers). Lane D-F. PCR on same samples using b-actin primers as internal control. M-100 bp ladder. (C) RNA in situ hybridization for otx1b in WT and Foxa1 morphant embryos. [file 1471-213X-11-63-S7.TIFF]
