# Supplementary material for: Conserved and non-conserved enhancers direct tissue specific transcription in ancient germ layer specific developmental control genes
Source: BMC Dev Biol. 2011 Oct 20;11:63. doi: 10.1186/1471-213X-11-63 (PMC3210094; doi:10.1186/1471-213X-11-63)
Supplement: Additional file 6 — Probe sequences used for EMSA and TRANSFAC details. Mutants probes used for EMSA and TRANSFAC score for putative transcription factors bound to the enhancer. [file 1471-213X-11-63-S6.DOC]

**CTCGTTCTCTGCCTTTCTCTTTCTTTCTTTCTCTCCCTCT** EM4

**AGATG**TCTCTGCCTTTCTCTTTCTTTCTTTCTCTCCCTCT Probe 1

CTCGT**GAGAG**GCCTTTCTCTTTCTTTCTTTCTCTCCCTCT Probe 2

CTCGTTCTCT**TAAGG**TCTCTTTCTTTCTTTCTCTCCCTCT Probe 3

CTCGTTCTCTGCCTT**GAGAG**TTCTTTCTTTCTCTCCCTCT Probe 4

CTCGTTCTCTGCCTTTCTCT**GGAGG**TCTTTCTCTCCCTCT Probe 5

CTCGTTCTCTGCCTTTCTCTTTCTT**GAGGG**CTCTCCCTCT Probe 6

CTCGTTCTCTGCCTTTCTCTTTCTTTCTTT**AGAGA**CCTCT Probe 7

CTCGTTCTCTGCCTTTCTCTTTCTTTCTTTCTCTC**AAGAG** Probe 8

**1) The nucleotides in red are the ones mutated (5 bp) with respect to their wild type sequence in EM4**

**CTCGTTCTCTGCCTTTCTCTTTCTTTCTTTCTCTCCCTCT** EM4

**AGATGGAGAG**GCCTTTCTCTTTCTTTCTTTCTCTCCCTCT M1-10

CTCGTTCTCT**TAAGGGAGAG**TTCTTTCTTTCTCTCCCTCT M11-20

CTCGTTCTCTGCCTTTCTCT**GGAGGGAGGG**CTCTCCCTCT M21-30

CTCGTTCTCTGCCTTTCTCTTTCTTTCTTT**AGAGAAAGAG** M31-40

**2) The nucleotides in red are the ones mutated (10 bp) with respect to their wild type sequence in EM4**

| **Sequence** | **TRANSFAC Score** | **Transcription Factor** |
| --- | --- | --- |
| **cttcTCTTTctttc** | **0.93** | **Foxa1** |
| **tctctttCTTTC** | **0.80** | **Pbx2** |
| **tCTTTCtttct** | **0.81** | **Lef1/Tcf1** |

**CTCGTTCTCTGCCTTTCTCTTTCTTTCTTTCTCTCCCTCT Foxa1**

**CTCGTTCTCTGCCTTTCTCTTTCTTTCTTTCTCTCCCTCT Pbx2**

**CTCGTTCTCTGCCTTTCTCTTTCTTTCTTTCTCTCCCTCT Lef1/Tcf1**

**3) Table showing the TRANSFAC motif score of the three potential transcription factors that can bind the region EM4. The 3 regions have been separately highlighted below.**
